# Pancytopenia in a Patient with Stevens-Johnson Syndrome: A Case Report with Literature Review

**DOI:** 10.7759/cureus.4702

**Published:** 2019-05-21

**Authors:** Mustafa N Malik, Ateeqa Mujeeb Ullah, Muhaddis Ejaz Ahmad, Rida Riaz, Tariq Iqtidar Sadiq Syed

**Affiliations:** 1 Internal Medicine, The University of Arizona, Tucson, USA; 2 Internal Medicine, Abwa Medical College and Research Center, Faisalabad, PAK; 3 Internal Medicine, Rawalpindi Medical University, Rawalpindi, PAK; 4 Internal Medicine, Nawaz Sharif Medical College - University of Gujrat, Gujrat, PAK; 5 Internal Medicine, Allama Iqbal Medical College, Lahore, PAK

**Keywords:** stevens-johnson syndrome, toxic epidermal necrolysis, pancytopenia, hemorrhagic pleural effusion, thrombocytopenia

## Abstract

Stevens-Johnson syndrome (SJS) and toxic epidermal necrolysis (TEN) are rare conditions triggered due to a medication that involves the necrosis and desquamation of the skin and mucous membranes. Only one out of 1,000,000 people are affected by the condition. The exact pathophysiology of the disease is still unknown. However, many complications of the disease can occur; pancytopenia and pleural effusion are an even rarer find. Here we present a case of a 17-year-old male who presented with fever and rash for 15 days associated with bleeding per rectum, hemoptysis, and conjunctival hemorrhages. Laboratory investigations showed severe pancytopenia, deranged liver function tests (LFTs), and hypocellular bone marrow. The patient started showing improvement after 10 days post-admission with supportive care and multiple transfusions.

## Introduction

Stevens-Johnson syndrome (SJS) and toxic epidermal necrolysis (TEN) can be categorized as severe delayed hypersensitivity drug reactions identified by widespread epidermal and mucocutaneous detachment [1-2]. The disease spectrum contains SJS on one end with less than 10% body surface area being covered by desquamated skin whereas TEN sits on the other extreme with more than 30% of the body surface area being involved. The window in between is a gray area known as a mix of the two [3]. The most common culprits associated with SJS/TEN include allopurinol, anticonvulsants (carbamazepine and lamotrigine), and sulfonamide antibiotics [1]. The incidence of SJS is said to be one to six per million people per year while that of TEN it is only 0.4-1.2 [4-5]. The average mortality rate for TEN is 25%-35% while for SJS it is 1%-5% [3]. Among the many complications that can occur due to these disorders, hematological abnormalities such as thrombocytopenia leading to a deranged coagulation profile are very uncommon [6]. The prevalence of pancytopenia as a unit only in SJS/TEN has a handful of reported cases [5]. We report a case of a 17-year-old male with SJS and severe pancytopenia. The most unique finding was thrombocytopenia, which leads to most of the complications in our patient.

## Case presentation

A 20-year-old male presented with a rash for 15 days followed by hemoptysis, bleeding per rectum, and conjunctival hemorrhages for seven days, and jaundice for four days, after he took a medication (sulfamethoxazole-trimethoprim salt) for fever from his primary care physician. The rash erupted on his trunk about half an hour after taking the medication. The rash was intensely pruritic and progressed to involve the whole body with no bulla formation, photosensitivity, burning micturition, or joint pains. The patient also had two episodes of hemoptysis after he experienced severe bouts of cough on lying straight. The patient also reported three episodes of bleeding per rectum which was fresh, red in color, without clots, not associated with defecation or straining. There was intense reddish discoloration of his eyes with no associated itching, watering, or pain. On further inquiry, the patient also reported yellowish discoloration of his skin and eyes. The fever was low grade, continuous in nature, undocumented, not associated with rigors/chills, retro-orbital pain, productive cough, or abdominal pain.

**Figure 1:**
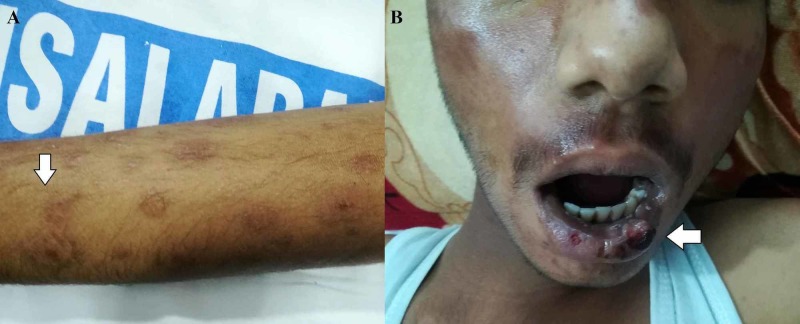
A) Widespread round to oval, purpuric rash covering the upper limb. B) Mild erosions with healing ulcerations covering the lips and the oral cavity.

On physical examination, there was visible pallor, jaundice, tachycardia (112 beats/minute), tachypnoea (24 breaths/minute), blood pressure (BP) of 110/70 mmHg, and temperature of 99 degrees Fahrenheit. Dermatological examination showed an erythematous rash involving the whole body with some areas of post-inflammatory hyperpigmentation markedly over the face and upper trunk. The upper limbs were covered with a purpuric rash, round to oval in shape (Figure 1A). The oral cavity and lips showed mild erosions with healing ulcerations (Figure 1B) and the eyes exhibited conjunctival congestion.

The genitalia, nails, and scalp were unremarkable. On chest examination, no visible deformities were present, breathe sounds were decreased on the left side while the right side of the chest had normal vesicular breathing.

**Figure 2:**
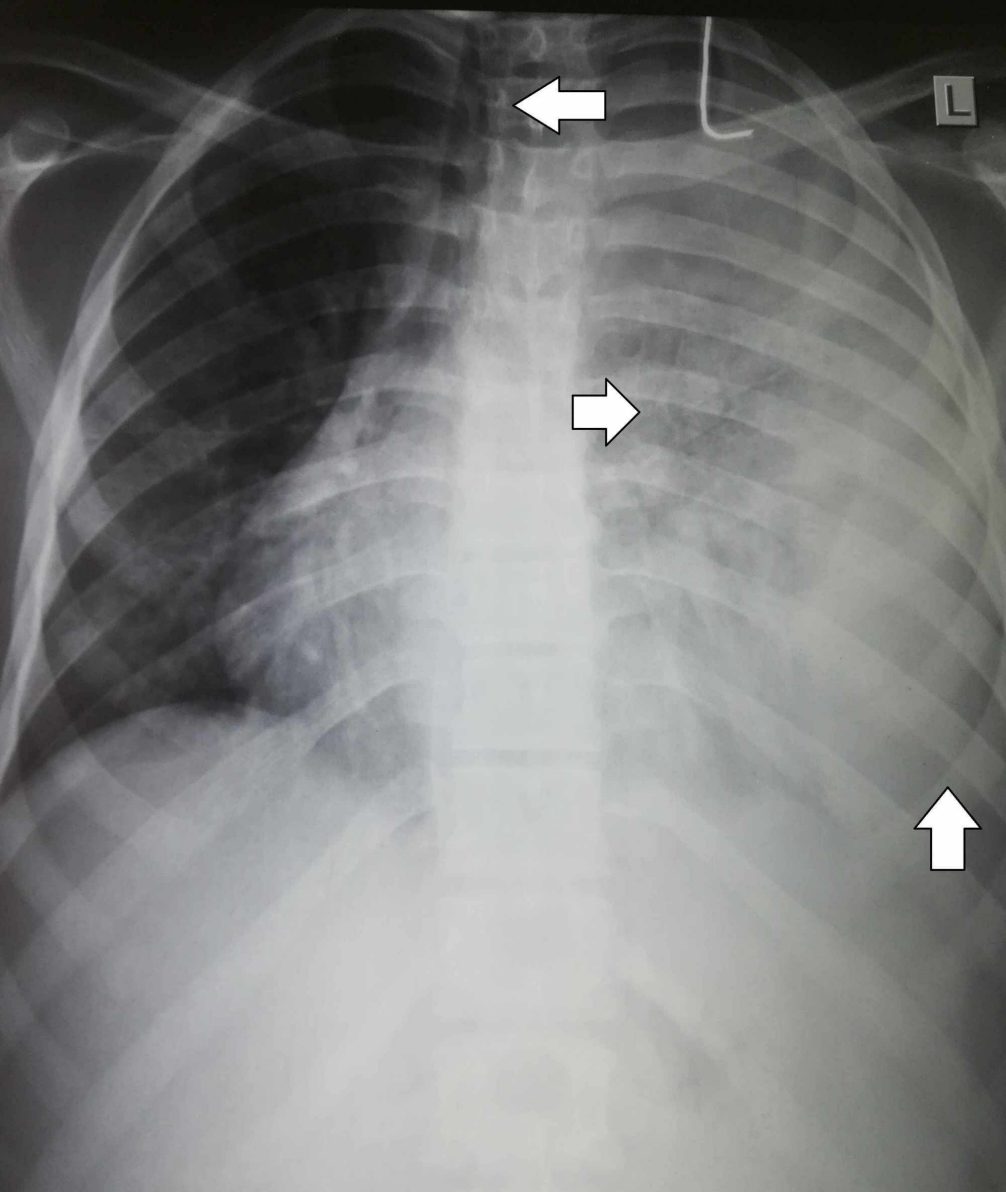
Chest radiograph showing left-sided pleural effusion with mild tracheal deviation to the right and prominent lung markings.

On laboratory investigations, hemoglobin was 4.2 g/dL, platelet count was 21,000/uL, and white blood cell (WBC) count was 600/uL (neutrophils 18%, lymphocytes 82%). Peripheral smear showed severe hypochromic, normocytic anemia with severe leukopenia, and thrombocytopenia. Reticulocyte count was normal. Bone marrow biopsy revealed hypocellularity. Erythrocyte sedimentation rate (ESR) was 162 mm/h. On coagulation profile, prothrombin time (PT) was 17 s whereas activated partial thromboplastin time (APTT) was 43 s. Liver function tests (LFTs) showed total bilirubin of 7.8 (direct bilirubin 4.8, indirect bilirubin 3.1), alanine aminotransferase (ALT) of 176, and aspartate transaminase (AST) of 142. Renal function tests were normal. Urine was positive for urobilinogen, bilirubin, and albumin. Chest radiograph showed a complete whitewash of the left lung with mild tracheal deviation to the right and prominent lung markings (Figure 2).

A diagnostic and therapeutic pleural tap was performed which was reddish in color and turbid in appearance. The chemical and cytological analysis of the pleural fluid showed proteins (5.3 g/dL), WBC count (3800/mm) (neutrophils 60%, lymphocytes 40%), RBC count showing numerous cells, no microorganisms or malignant cells were seen, and acid-fast bacilli (AFB) stain was negative. Abdominal ultrasonography was unremarkable.

The patient received supportive care including appropriate wound care, eye, and oral care and pain relief. Six pints of red blood cells were transfused along with two pints of fresh frozen plasma. The patient also received injection erythropoietin and injection transaminase. He started to show improvement at the 10th post-admission day supported by laboratory parameters. He was discharged on the 15th post-admission day with weekly follow-ups.

## Discussion

Stevens-Johnson syndrome (SJS) and TEN are a part of the spectrum of serious dermatological manifestations of drugs and can become life-threatening conditions in most instances [7]. These two conditions are differentiated based on the percentage of the body surface area affected, with SJS affecting less than 10% [2]. SJS was initially categorized by Stevens and Johnson in 1922 in two children as “extraordinary, generalized eruption with continued fever, inflamed buccal mucosa and severe purulent conjunctivitis” [8]. Common etiologies include: A) Drugs: allopurinol (used for gouty arthritis); antiepileptic drugs (carbamazepine and lamotrigine); antibiotics (mostly sulfa containing); non-steroidal anti-inflammatory agents (NSAIDs) in some instances [2, 9]; B) The infectious etiology of these reactions is very minor compared to the drug aspect but most commonly associated are multiple viruses from the Herpes family and Mycoplasma pneumoniae (especially in children) [2, 8].

The initial symptoms start with fever, burning of eyes, and difficulty swallowing followed by erythematous, dusky-red or purpuric macules varying in shape and size, starting at the trunk and moving outwards. The palms and soles are also involved. All mucous membranes have a tendency of being involved, with buccal, ocular, and genital mucosas more commonly while respiratory and gastrointestinal mucosas in rare instances. The most common early complications include superficial skin infections, sepsis, and pneumonia while long-term sequelae are categorized into skin (pruritis, hypertrophic scars), ocular (dry watery eyes, decreased visual acuity) and organ-specific (vaginal/labial adhesions, bronchiolitis obliterans) (Table 1) [2, 7, 10].

**Table 1:** Complications associated with Stevens-Johnson syndrome and toxic epidermal necrolysis (SJS/TEN).

Early complications	Late complications [2, 7, 10]
Secondary skin infections	Immunological (idiopathic thrombocytopenic purpura)
Oral and gastrointestinal tract ulcerations	Vaginal and labial adhesions
Septicemia	Gastrointestinal adhesions
Hepatitis	Bronchiolitis obliterans
Pneumonia acute respiratory failure	Cutaneous (dyschromic macules, hypertrophic scars, hair loss, nail dystrophy)
Hematological (anemia, leukopenia, leukocytosis with neutrophilia, thrombocytopenia)	Ocular (decreased visual acuity, corneal scarring, photophobia with watery eyes, keratoconjunctivitis sicca, keratitis, entropion, adhesion, complete blindness)

Recent studies suggest that cytotoxic granules produced by cytotoxic T-cells mediate skin damage in SJS and TEN. However, the exact pathogenic mechanism of skin damage in SJS/TEN is not fully understood. The most severe skin damage from acute graft-versus-host disease (GVHD) after hematopoietic stem cell transplantation (HSCT) is clinically and histologically similar to TEN. Therefore, it can be concluded that animal models of acute GVHD can reveal details about T-cell-induced severe cutaneous adverse reactions such as SJS/TEN. Azukizawa in his review discussed the pathogenesis of SJS/TEN in reference to animal models of keratinocyte-specific skin diseases that are similar to acute GVHD. These models demonstrated that the essential effector cells involved in the pathogenesis of both SJS and TEN are CD8+ cytotoxic T-cells, and for the pathogenesis of TEN, the loss of suppression by thymus-derived, regulatory T-cells is crucial [11-12].

The characteristic that separates SJS and TEN from other cutaneous drug eruptions is the detachment of the epidermis from the underlying dermis which also forms the basis of diagnosis as the diagnosis is mainly based on clinical and symptomatic grounds. The Nikolsky sign is a usually used parameter for clinical diagnosis of SJS/TEN and is described as the separation of the superficial skin when side-ways pressure is applied to an area of erythematous nonblistering skin [2-3]. Laboratory parameters that can be deranged in SJS/TEN include lymphopenia, anemia, elevated erythrocyte sedimentation rate (ESR) and LFTs, and in rare instances, thrombocytopenia but the definitive diagnosis can only be made on skin biopsy [2, 8]. Necrotic keratinocytes, epidermal-dermal junction changes, dermal infiltrate, and edema in the papillary dermis with or without eosinophils are the most common findings on a biopsy [13]. However, due to its invasive nature and a long, tedious procedure, it is not commonly used as a diagnostic tool [2].

No set criterion of drug monotherapy or drug combination has yet been proven to be efficacious in treating these spectra of diseases [14]. The management strategy still largely employed is a multidisciplinary approach starting from prompt drug withdrawal to supportive treatment, e.g. aggressive wound care, infection control, ocular and gastrointestinal tract care, high protein diet, and whole blood or separate component transfusions [1, 15]. Corticosteroids, though a common practice in some countries, has not yet been established as a definitive treatment for SJS worldwide [2]. In a 10-year retrospective cohort study by Chan and Cook et al., 42 patients were included. Out of these 42 patients, 26 (62%) patients had TEN, six (14%) patients had SJS/TEN overlap, and 10 (24%) patients had SJS. Thirteen patients received combination therapy with intravenous immunoglobulin (IVIG) and systemic corticosteroid; seven patients received systemic corticosteroid monotherapy, 16 patients received IVIG monotherapy; and six patients received supportive therapy alone. No deaths were reported in the combined group, two deaths in the corticosteroid group, five deaths in the IVIG group, and one death was reported in the supportive therapy group. On comparing active treatment groups, a statistically significant survival benefit with combination therapy was reported (p = 0.05). There is a need for randomized prospective cohort studies involving larger patient populations to further explore the efficacy of combination therapy with IVIG and corticosteroids in SJS/TEN patients [16].

The prognosis of these drug reactions has improved considerably over the past few years due to a better understanding of the pathobiology which includes a genetic link to human leukocyte antigen (HLA) and non-HLA genes, T-cell mediated cytotoxicity against metabolites of the drug, and T-cell receptor (TCR) restriction [9, 15]. However, the prognostic window still remains poor due to the lack of prompt diagnosis and intensive supportive and nutritional care needed thereafter. The poor prognosis can also be attributed to the fact that these reactions are caused by a set of commonly used drugs [9, 17].

Pancytopenia in patients with SJS/TEN starts with severe lymphopenia which can be due to direct antigen reaction with the cells or antigens affecting bone marrow functions and is the most common hematological abnormality seen in these patients [6]. This can render the patient susceptible to many infections, the most commonly encountered of which are superficial bacterial skin infections, pneumonia, and sepsis [2]. Our patient remained infection free owing to rigorous wound care and supportive therapy. Thrombocytopenia in SJS is an uncommon phenomenon and the mechanisms although theorized are poorly understood [6, 18]. In our case, thrombocytopenia and direct/indirect drug-induced liver insult caused the coagulation cascade to become askew. This led to a left-sided hemorrhagic pleural effusion, hemoptysis, and episodes of bleeding per rectum. A direct bone marrow insult plus bleeding from different sites led to severe anemia in our patient. Although severe pancytopenia is considered an association of other more lethal immune disorders when occurring with SJS but no such association was found in our patient based on diagnostic guidelines [19].

## Conclusions

Stevens-Johnson syndrome with pancytopenia is a clinical rarity and not much research has been conducted into the disease thus far. Owing to the nature of the disease, its rapid onset, progress, positive mortality index and the toll it takes on the health and nutrition of the patient, it can be safely concluded that association of SJS/TEN combined with leucopenia, thrombocytopenia, and anemia poses an even greater life risk. Supportive management like appropriate infection control, fresh frozen plasma and red cell concentrate transfusions, injection transaminase and erythropoietin are often life-saving in such cases.
